# *Strongyloides*: omics to worm-free populations

**DOI:** 10.1098/rstb.2022.0448

**Published:** 2024-01-15

**Authors:** Dora Buonfrate, Vicky L. Hunt, Peter Odermatt, Adrian Streit

**Affiliations:** ^1^ IRCCS Sacro Cuore Don Calabria hospital, Negrar, Verona 37024, Italy; ^2^ Department of Life Sciences, University of Bath, Bath BA2 7AY, UK; ^3^ Epidemiology and Public Health, Swiss Tropical and Public Health Insitute, Allschwil, Basel 4123, Switherland; ^4^ Max Planck Institute for Biology Tübingen, Tübingen, Baden-Württemberg 72076, Germany

**Keywords:** *Strongyloides*, soil-transmitted helminth, Neglected Tropical Disease, nematode, parasite

## Abstract

This article is part of the Theo Murphy meeting issue ‘*Strongyloides*: omics to worm-free populations’.

The nematode genus *Strongyloides* consists of more than 50 different species that are all small intestinal parasites of vertebrates other than fish [1] and are of variable veterinary and medical importance [2–5]. The threat for human health caused by the species *Strongyloides stercoralis* is increasingly appreciated [3–5] after it had been grossly neglected for a long time. Human strongyloidiasis is included in the WHO list of the Neglected Tropical Diseases (NTD) [6,7] and the estimated number of people infected with *S. stercoralis* has recently been raised to about 600 million people [3]. The prevalence of *S. stercoralis* was, and probably still is, underestimated since specific diagnostic methodology is required and all such methodology has issues with sensitivity and/or specificity [8–10]. Another reason that infections are frequently missed is that, although *S. stercoralis* infections can be fatal, most infections show only mild or no clinical symptoms and if there are symptoms, they are rather unspecific [4]. *Strongyloides stercoralis* has a cosmopolitan distribution but is strongly enriched in tropical and subtropical socioeconomically disadvantaged regions [3,11]. The recommended treatment for *S. stercoralis* is ivermectin, which is highly effective but unfortunately not available in all countries. Mass drug administration (MDA) is under evaluation by the World Health Organization (WHO) to control strongyloidiasis in endemic areas [4,12].

*Strongyloides* spp. is also an emerging model system for translational, basic biological and evolutionary research [13–17]. Both the medical threat that it poses and its attractiveness for basic research are connected to its rather complicated life cycle. The life cycle of *Strongyloides* spp. has been reviewed repeatedly (e.g. [16,18,19]) and is summarized here (figure 1). AlI infective third-stage larvae (iL3) are females and they enter a new host by skin penetration. After migrating through the blood and the lungs or nose (dependent on the species), the larvae are swallowed and eventually reach the small intestine of the host where they complete their development to parthenogenetically reproducing parasitic adults. Whether alternative migration paths through the host's body are also possible is a matter of debate (see article by Al-Jawabreh and colleagues [] in this special issue). Dependent on the species, the progeny of the parasitic females have three or four developmental options. 1) They may become female, leave the host as embryonated eggs or first-stage larvae (dependent on the species), develop in the environment into iL3 and search for a new host (called direct or homogonic development), closing an asexual reproductive cycle. 2) They may become female and leave the host as embryonated eggs or first-stage larvae but develop into free-living, non-infective third-stage larvae and subsequently into adult females (indirect or heterogonic development). 3) They may become male and leave the host as embryonated eggs or first-stage larvae and develop into free-living adult males (indirect or heterogonic development). The free-living adults mate and reproduce in the environment and all their progeny are females and develop to iL3s, completing a sexual reproductive cycle (as the only exception, *S. planiceps* has been described to be capable of undergoing up to nine consecutive free-living generations [21]). 4) They may become female, and develop into autoinfective third-stage larvae (aiL3) within the host and re-infect the same host individual (autoinfective cycle, asexual). While all species of *Strongyloides* (but not necessarily all isolates of these species [22–24]) may undergo homogonic or heterogonic development, the autoinfective cycle (option 4) appears to be specific for *S*. *stercoralis* and maybe a few other less well-investigated species [18]. The species-specific existence of this autoinfective cycle is the reason why strongyloidiasis is a serious threat to human health [3–5] but only of moderate veterinary concern, except for animals—such as dogs and monkeys—that can also carry *S. stercoralis* [2]. The autoinfective cycle allows the parasite to persist in an individual host for much longer than an individual worm can live outside a host (chronic strongyloidiasis). Usually, healthy individuals tolerate chronic infections well and control them at very low worm burdens [4]. Because such people have only mild or no symptoms and the worm burdens are so low, much of the routine parasitological diagnostic methodology is not suitable to detect *S. stercoralis* and chronic strongyloidiasis goes frequently unnoticed [9]. However, if a chronically infected patient becomes immunodeficient due to disease or immunosuppressive treatment (i.e. steroids, cancer chemotherapy or organ transplantation), the control of the autoinfective cycle may fail, leading to hyperinfection syndrome and disseminated strongyloidiasis, which are usually lethal if not treated in time due to late recognition and/or uncertainty about the best treatment strategy [4].

**Figure 1.**
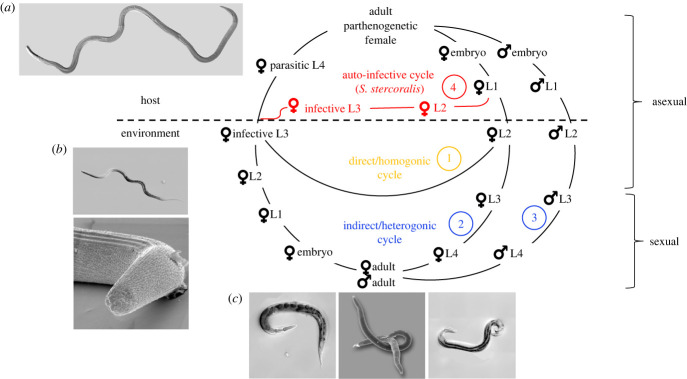
Life cycle of *Strongyloides stercoralis*. For explanations see text. The circled numbers refer to the developmental option numbers in the text. This life cycle also applies, with small modifications, to other species of *Strongyloides*. i.e. the autoinfective cycle appears specific for *S. stercoralis*; in some species the young larvae hatch while still in the host, while in other species embryonated eggs are passed; in *S. planiceps* multiple consecutive free-living generations are possible (for more information and references see text). The images show *Strongyloides papillosus* (*a*) adult parasitic female (top left): in this differential interference contrast (DIC) image, the worm is about 5 mm long (the size of adult females varies between species of *Strongyloides*; in *S. stercoralis* they are about 2.5 mm [1]); (*b*) infective L3 (bottom left): DIC image (upper panel) and scanning electron microscopic (SEM) image (lower panel); the worm is about 0.6 mm long; (*c*) free-living adults (bottom), DIC image of a female (left panel), DIC image of a male (right panel), SEM image of a mating couple (middle panel). The free-living adults are about 1 mm long.

For the basic biologist, *Strongyloides* spp. is an attractive system because of the availability of a free-living sexual generation of adults that provides, for a parasite, a quite unique opportunity for experimental manipulation, combined with a short generation time of a few days to a few weeks, dependent on the species [14,15]. A further advantage of *Strongyloides* spp. is the small size of the genome for members of this genus [25], Kounosu *et al.* this issue [26]). Several species of *Strongyloides* can be maintained in the laboratory relatively easily, either in their natural hosts (*S. ratti* and *S. venezuelensis* in rats [14]) or in permissive laboratory hosts (*S. papillosus* in rabbits [27] and *S. stercoralis* in dogs or gerbils [17,28]). While *S. ratti* and *S. venezuelensis* in particular provide attractive animal models to study *Strongyloides* biology in their natural host [14], the absence of the auto-infective cycle limits the study of pathogenicity in these species such that studies on the human pathogen itself are indispensable.

We had long felt that there are insufficient interactions between more applied, health-care oriented *Strongyloides* researchers and basic biologists working with this group of parasites and that both sides could profit from the expertise of the other. We had entertained the idea for a joint meeting for quite some time. Finally, the Royal Society enabled us to organize a Theo Murphy meeting entitled ‘*Strongyloides*: omics to worm-free populations’. On 28th and 29th November 2022 about 50 people interested in *Strongyloides* spp., including clinicians, diagnosticians, epidemiologists, geneticists, molecular biologists, bioinformaticians and immunologists, met in in Frome, England to discuss the biology and the control of *Strongyloides* spp., with a strong emphasis on *S. stercoralis*.

*Philosophical Transactions B* offered to publish a special issue related to the Theo Murphy Meeting and invited us, the scientific organizers of this conference, to guest edit it. Twelve papers were accepted for publication in this special issue. They are briefly mentioned below.

Overall, we have to admit that, although *Strongyloides* spp. has been studied for more than 160 years, there are still substantial gaps in our understanding of these parasites, some of which are almost embarrassing because they concern very basic aspects of *Strongyloides* biology and pathogenicity. For the first article in this special issue, to which all meeting participants were invited to contribute, Mark Viney compiled a list of open questions in *Strongyloides* biology, immunology, pathogenesis, diagnostics and control (Al-Jawabreh *et al*. [20]).

Dora Buonfrate, Antonio Montresor, Zeno Bisoffi, Francesca Tamarozzi and Donal Bisanzio estimate the global number of adults who should be included in MDA for strongyloidiasis, which could be used by endemic countries to calculate sources and funds needed to implement control programmes (Buonfrate *et al.* [29]).

Pockets of poverty can lead to disproportionately high prevalence of strongyloidiasis even in populations living in one of the world's wealthiest countries, Australia. Kirstin Ross describes vividly the issues leading to high strongyloidiasis rates in First Nation communities, and advocates for action to fight this situation (Ross [30]).

Benjamin Collyer and Roy Anderson present a stochastic individual-based model that is aimed at evaluating the impact of MDA for strongyloidiasis, although some knowledge gaps (e.g. dynamics of post-treatment re-infection) still limit its application (Collyer & Anderson [31]).

It had already been noticed in very early reports about the human-infective *S. stercoralis* that dogs carry *Strongyloides* spp. that are similar to the human ones. This followed a decade-long discussion over whether the *Strongyloides* spp. in dogs is the same or just very similar to the one in humans—and with this, if dogs are a reservoir for zoonotic strongyloidiasis. In their article, Richard Bradbury and Adrian Streit discuss this issue, which is still not resolved (Bradbury & Streit [32]).

Eva Nosková, Kelly Sambucci, Klara Petrzelkova, Barbora Cervena, David Modry and Barbora Pafco discuss *Strongyloides* infections in humans and non-human primates, and highlight gaps in the currently available data and the importance of this information for understanding zoonosis transmission and pathogenicity (Pafko *et al*. [33]).

A crucial step in the life cycle of *Strongyloides* is finding and percutaneously entering a host individual. Courtney McClure, Ruhi Patel and Elissa Hallem review the current knowledge of skin-penetration behaviour and the underlaying mechanisms for *Strongyloides* and for hookworms, which are phylogenetically rather distant nematode parasites with similar infection biology (McClure *et al*. [34]).

In their article, Minka Breloer and Lara Linnemann review what is known about the immune response that *S. ratti* and *S. stercoralis* elicit in their natural hosts and in mice that are permissive laboratory hosts, and they provide the unique tools of mouse genetics and immunology to the study of *Strongyloides* infection biology. The authors also discuss the strategies that the parasite employs to cope with the host's defence mechanism (Breloer & Linnemann [35]).

In the next contribution, Reem Al-Jawabreh, Dominika Lastik, Darrin McKenzie, Kieran Reynolds, Mona Suleiman, Angela Mousley, Louise Atkinson and Vicky Hunt discuss the state of -omics data and resources for *Strongyloides* spp. and compare them to the model nematode *Caenorhabditis elegans* (Al-Jawabreh *et al*. [36]).

Asuka Kounosu, Simo Sun, Yasunobu Maeda, Mehmet Dayi, Akemi Yoshida, Haruhiko Maruyama, Vicky Hunt, Asako Sugimoto and Taisei Kikuchi report chromosomally complete or near complete genome assemblies of two species of *Strongyloides* with different numbers of chromosomes (*S. ratti* and *S. venezuelensis*) and *Rhabditophanes diuinus* the phylogenetically closest non-parasitic relative of S*trongyloides* spp. currently known. They investigate the syntenic relationships and discuss the genome evolution in these species (Kounosu *et al*. [26]).

Natalia Tiberti, Marcello Manfredi, Chiara Piubelli and Dora Buonfrate discuss *Strongloides* proteomics data and present results for the first study on serum proteomics from patients suffering from strongyloidiasis (Tiberti *et al*. [37]).

Astra Bryant, Damia Akimori, Jonathan Stoltzfus and Elissa Hallem highlight gene annotation errors in the *Strongyloides* genomes and present a workflow for improving gene annotations and correcting errors (Bryant *et al*. [38]).

## Data Availability

This article has no additional data.
